# Unilateral Angiokeratoma of Fordyce With Unilateral PEAKER: A Rare Presentation of an Uncommon Disease

**DOI:** 10.7759/cureus.28926

**Published:** 2022-09-08

**Authors:** Ashwin Vinod, Pradeep Jacob, Ajith T Nair, Ranjith V B

**Affiliations:** 1 General Surgery, Amrita Institute of Medical Sciences and Hospital, Cochin, IND

**Keywords:** diagnostic dilemma, cryotherapy, surgical excision, penile angiokeratoma, fordyce angiokeratoma

## Abstract

Angiokeratomas are benign vascular lesions that can occur anywhere in the body. Fordyce angiokeratomas, also known as genital angiokeratomas, often develop on the vulva in women and the scrotum in males. A subtype of genital angiokeratoma in males is called penile angiokeratoma (PEAKER). In females, clitoral angiokeratoma (CLANKER) is the embryologic equivalent. As a result of the underlying pathophysiology, these lesions are often bilateral. Unilateral Fordyce angiokeratoma instances are infrequent, and unilateral PEAKER cases have never been previously documented. We describe a case of Fordyce's unilateral angiokeratoma with unilateral PEAKER. To the best of our knowledge, such a variation in presentation of this rare disease has not been previously reported.

## Introduction

Angiokeratomas are benign vascular lesions distinguished by dilated superficial blood vessels and underlying hyperkeratosis. Imperial and Helwig developed a taxonomy of angiokeratoma in February 1967 [1]. Angiokeratoma corporis diffusum (Fabry's disease), angiokeratoma of Mibelli, angiokeratoma of Fordyce, angiokeratoma circumscriptum neviforme (ACN), and solitary or multiple angiokeratomata are the five clinical kinds of angiokeratoma. John Addison Fordyce first described Fordyce angiokeratoma, also known as genital angiokeratoma, in 1896 [2]. A 60-year-old man hospitalized with urinary issues was the first person to disclose having genital angiokeratoma. Dr. Fordyce discovered several tiny, dark purple lesions on his scrotum with round shapes. An angiokeratoma was found in a skin biopsy taken from one of the lesions. Angiokeratoma of the penis (PEAKER), a subtype of genital angiokeratoma, was first reported by Imperial and Helwig in their study on angiokeratoma in 1967 [1]. Genital angiokeratomas occur either on the scrotum or on the penis in isolation. As a result of the underlying pathophysiology, these lesions are often bilateral. Unilateral Fordyce angiokeratoma instances are infrequent, and unilateral PEAKER cases have never been previously documented. The occurrence of scrotal angiokeratoma along with PEAKER is uncommon. To the best of our knowledge, no previous case of unilateral Angiokeratoma of Fordyce with unilateral PEAKER has been reported.

## Case presentation

**Figure 1:**
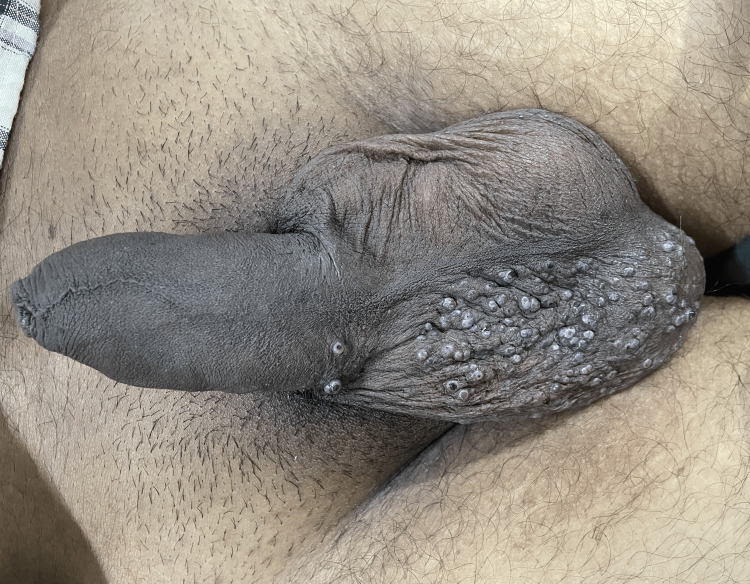
Clinical appearance of the lesions Multiple red-purple papules affecting only the right side of the scrotum and the penis. Left side is unaffected.

A 30-year-old man, who had developed several asymptomatic cutaneous lesions on his scrotum over three years, presented to our hospital due to a rapid increase in the size of the lesions. His medical background showed no significant comorbidities. Upon examination, several red-purple papules with a diameter of 2 to 3 mm, which only affected the right side of the scrotum and did not cross the midline, were found. Two similar lesions were noted on the right side of the penis, which did not cross the midline either (Figure 1). The angiokeratoma of Fordyce and the angiokeratoma circumscriptum neviforme (ACN) were part of the clinical differential diagnosis.

**Figure 2:**
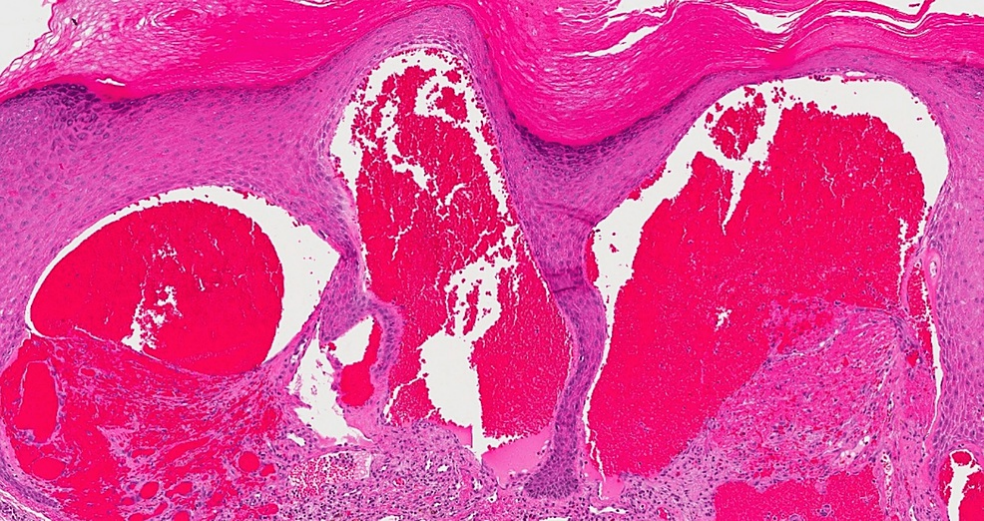
Histopathological examination image Hematoxylin and Eosin stained slide at 25x magnification showing dilated blood vessels in the papillary dermis, ectatic vessels in intimate contact with the epidermis and the presence of focal thrombus.

**Figure 3:**
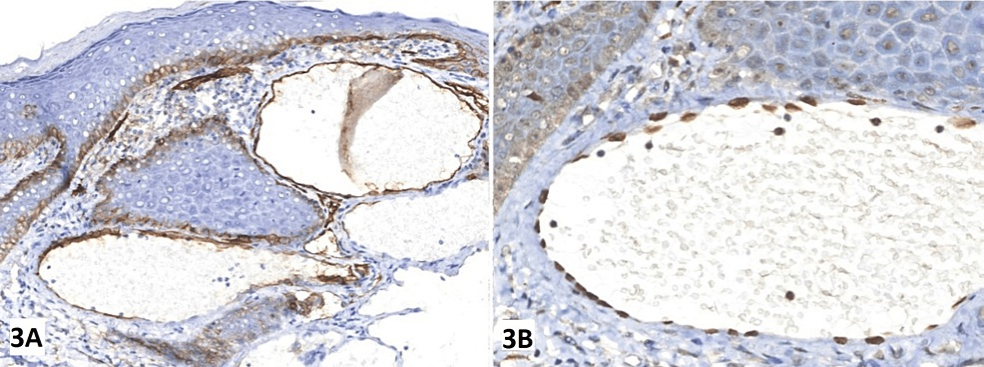
Immunohistochemical staining with CD31 Immunohistochemical (IHC) staining for CD31 revealed that the endothelial cells lining the dilated vascular structures were positive for CD31. 3A - IHC slide with original magnification at 100x. 3B - IHC slide with original magnification at 250x.

Dilated blood vessels were seen in the papillary dermis of the biopsy specimen of the lesions from the scrotum and penis. The epidermis was in close touch with these ectatic veins, and they had focal thrombosis (Figure 2). According to the immunohistochemical analysis, the endothelial cells that bordered the dilated vascular structures stained positively for CD31 (Figure 3). The diagnosis of unilateral angiokeratoma of Fordyce and unilateral PEAKER was determined based on the patient's age at presentation and the clinical and histological characteristics. Routine blood investigations were within normal limits. There were no apparent abnormalities seen during the abdominal, pelvic and scrotal ultrasonography.

**Figure 4:**
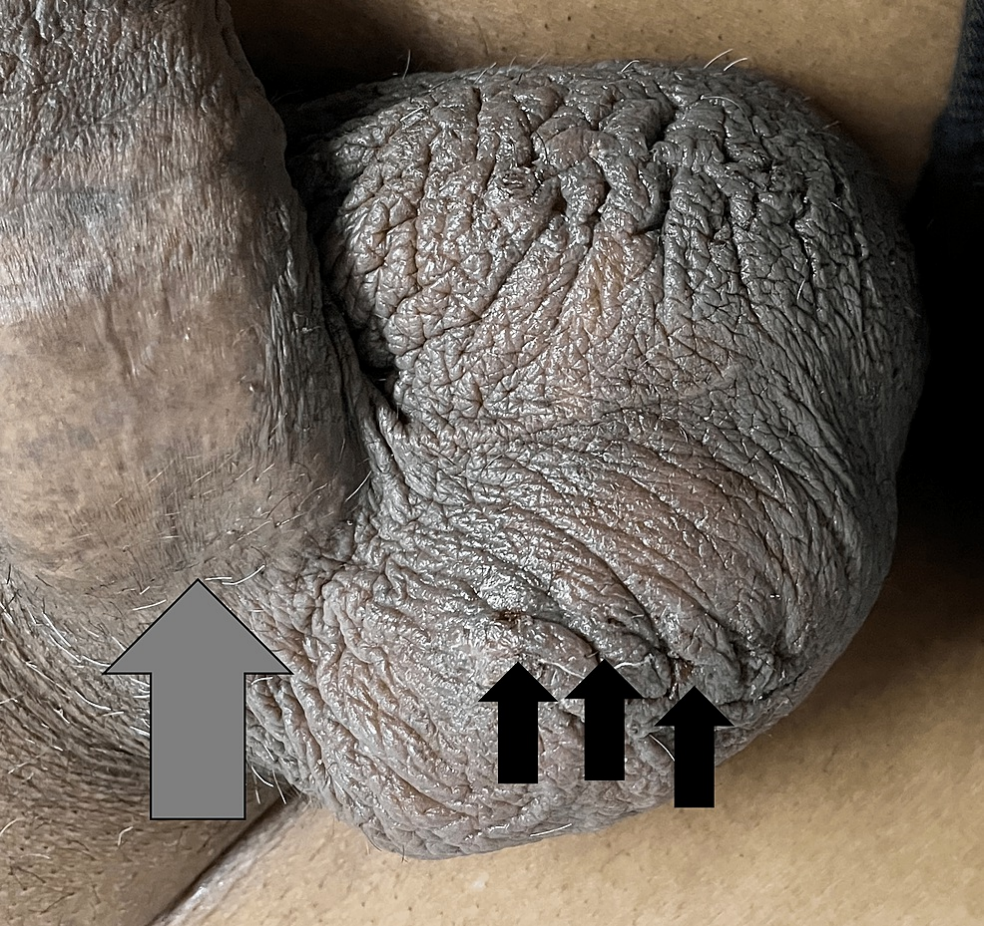
Clinical image at 12 weeks follow-up Grey Arrow - Site of PEAKER; Shows complete healing with no scarring following cryotherapy. Black Arrows - Sites of Angiokeratoma of Fordyce; Shows complete healing with only 1-2 lesions showing very minimal scarring.

The patient was treated with cryotherapy and showed an excellent clinical response to treatment with all lesions resolving without scarring or recurrence at 12 weeks of follow-up (Figure 4).

## Discussion

Although it can occasionally appear in infancy or adolescence, angiokeratoma of Fordyce primarily manifests in late adulthood or older adults. The lesions are often bilateral, numerous, and occur on the scrotum. However, they can occasionally be detected on the thighs, buttocks, belly, or shaft or glans of the penis [3]. Scrotal angiokeratoma's pathophysiology has been associated with a number of conditions; however, the exact etiology is still unclear. Some of the postulated theories include - a decline in the elasticity of the venous supporting tissues, a direct insult to the dermal capillaries, and persistent, localized scrotal venous hypertension [4,5].

Regarding the last point, scrotal angiokeratoma may occur as a result of certain disorders that might raise the blood pressure in the scrotal veins, including hernias, epididymal or urinary tract tumors, and notably varicoceles.

Unilateral angiokeratoma of the scrotum has been very rarely reported. Only five cases of unilateral scrotal angiokeratoma have been documented in the literature [6]. Also, only 54 occurrences of the uncommon but unusual genital angiokeratoma subtype PEAKER have been documented so far [7].

We want to draw attention to some significant differences by contrasting our case with those previously reported. In all prior cases, angiomatous lesions were associated with a varicocele [6]. Three of the five patients with unilateral angiokeratoma of the scrotum had lesions confined to the left side of the scrotum and linked with a left-sided varicocele [6]. In all the previously reported cases of PEAKER, the lesions were bilateral, involving both sides of the glans, corona, or shaft of the penis [7].

However, in our case, the angiokeratomas were peculiarly isolated to the right scrotum and unrelated to a varicocele. Given that varicoceles are relatively prevalent, the correlation of angiokeratomas with them in the above examples may be coincidental. However, it is also probable that the ipsilateral varicocele in individuals with unilateral angiokeratomas and angiomatous lesions contributed to their higher venous pressure. Also, the lesions over the penis did not cross the midline, as seen in all other previously reported cases.

It was unclear why the angiomatous lesions in our case manifested unilaterally. Mosaicism, which would limit the development of angiokeratomas to genetically sensitive regions, might be one explanation. In this regard, it has been hypothesized that, at least in some cases, scrotal angiokeratomas may result from a congenital abnormality affecting the walls of the venules [8]. However, the lesions do not manifest themselves clinically until maturity. ACN was the primary differential diagnosis in our case, despite the fact that it often affects the lower extremities and is present from birth [9]. PEAKER has to be differentiated from various conditions, particularly angiokeratoma corporis diffusum [7]. Finally, another hypothesis is that the development of the vascular lesions specific to the right scrotum was brought on by an earlier harmful event (such as persistent irritation).

The treatment options include excision, curettage and cautery, radiofrequency cautery, sclerotherapy, cryotherapy, and laser ablation. Among these, the potassium-titanyl-phosphate (KTP) laser and the 800 nm diode laser are considered to generate the most negligible scarring [7]. For the patient discussed in this article, the usage of cryotherapy has shown excellent clinical outcomes. Males with PEAKER, an uncommon and potentially asymptomatic type of genital angiokeratoma, may be treated conservatively [7]. Any surgical treatment method, including laser ablation, may be used to treat symptomatic individuals and those who experience anxiety and psychological distress due to the lesions’ form, location, or complications.

## Conclusions

The article discusses a rare mix of clinical manifestations of an uncommon illness. The treating surgeon or dermatologist has always faced a clinical conundrum when treating patients with scrotal and penile angiokeratomas. The patient in this report showed a unilateral angiokeratoma of Fordyce with unilateral angiokeratoma of the penis, without any underlying comorbidities. Such a presentation of the disease is extremely rare. Additionally, this report demonstrates that correctly administered cryotherapy is a successful way to treat this illness.

With this report, we aim to add to the existing literature and expand the spectrum of presentation of genital angiokeratoma, thus enabling a more accurate clinical diagnosis and effective treatment of the illness.
